# 

**Published:** 1988-11

**Authors:** 


					W1
NO,
br 3:
4
v* 103 au.4 1988
C.D1 SKQ; 333740000
BRISTOL ME'BICO?CHIRURGICAL
JOURNAL
VOLUME 103(iv)
NOVEMBER 1988
THE MEDICAL JOURNAL OF THE SOUTH WEST
250 YEARS OF PATIENT CARE IN
LTHE BRISTOL ROYAL INFIRMARY.
Preventive Medicine in General Practice.

				

